# Histopathological, Ultrastructural, and Immunohistochemical Assessment of Hippocampus Structures of Rats Exposed to TCDD and High Doses of Tocopherol and Acetylsalicylic Acid

**DOI:** 10.1155/2015/645603

**Published:** 2015-03-24

**Authors:** Joanna Rosińczuk, Robert Dymarek, Ireneusz Całkosiński

**Affiliations:** Department of Nervous System Diseases, The Faculty of Health Science, Wroclaw Medical University, Bartla 5 Street, 51-618 Wrocław, Poland

## Abstract

The effect of 2,3,7,8-tetrachlorodibenzo-p-dioxin (TCDD) on central nervous system consists of changing expression of estrogen receptors, whereas the result of chronic inflammatory reaction caused by dioxin is occurrence of destructive changes in various organs connected with disturbed metabolism of connective tissue and damage of cells. The aim of the study was to determine the effect of dioxins on function, ultrastructure, and cytological and histological structure of hippocampus, particularly on expression of estrogen receptors in central nervous system as well as to define protective influence of tocopherol (TCP) and acetylsalicylic acid (ASA) on the decrease in activity of proinflammatory effects in central nervous system. It was shown that TCDD contributes to destructive and inflammatory changes along with demyelization of myelin sheaths and atrophy of estrogen receptors in hippocampus. Dioxin contributes to atrophy of estrogen receptors in hippocampus, in which also destructive and inflammatory changes were found along with demyelination of myelin sheaths. Histopathological and ultrastructural image of hippocampus areas in rats, in which both TCP and ASA were used, is characterized by poorly expressed degenerative changes and smaller inflammatory reactivity. Using both TCP and ASA has a protective effect on functions of central nervous system.

## 1. Introduction

In the world literature there are not too many studies discussing the problem of dioxin effect on the function of central nervous system (CNS); however there are some hypotheses that the influence of 2,3,7,8-tetrachlorodibenzo-p-dioxin (TCDD) on CNS consists of changing expression of estrogen receptors.

It is justified by necessity for examinations of CNS oriented at reaction of oxidation stress and blockade of cyclooxygenase 2 (COX-2) and defining disorders of the function of estrogen receptors in CNS [1]. It was stated in the studies by Ishida et al. [2] that concentration of dioxins in brain of female rats which were administered with TCDD and in their offspring was comparable; however dispersion of TCDD in brain of fetuses calculated in relation of brain mass towards body mass was 100-fold bigger than in their mothers. The studies by Teraoka et al. [3] showed that TCDD stimulates COX-2 in CNS and because of the increase of prostaglandin concentration circulatory failure in brain occurs, which results in functional disturbances and destructive changes.

Subchronic exposure to TCDD contributes to diverse location of biogenic amines and related production of peroxide anions (ROS) in various areas of rat brain and causes neurotoxic effects of free radicals activity [4]. Molecular mechanism of dioxin action in brain has not been fully examined; therefore interest has been aroused to this subject matter. An important participant in the process of toxic activity of dioxins is aryl hydrocarbon receptor (AhR), which regulates transcription of various genes through binding with xenobiotic response element (XRE) at a fragment of DNA in nerve cells of CNS. AhR may play a crucial role in development of dioxin neurotoxicity and penetrating through the blood-brain barrier [5]. It was stated that dioxins passing through the blood-brain barrier and acting through AhR contribute to formation of free oxygen species. This receptor can be blocked by resveratrol. Moreover it was stated that the effect of dioxins on CNS might be limited by removing them from a cell by P-glycoproteins, if they are not connected with AhR [6].

Dioxins, which got to organism through alimentary tract and then passed from blood to liver, form junctions with aryl hydrocarbon AhR in cytosol of liver cells [7, 8]. This receptor reveals a very big similarity in activity mechanism to estrogen receptor (ER*α*) causing disorder of steroid hormone balance [9].

On the basis of latest literature reports, including our own research [1, 10], proinflammatory, multifaceted activity of dioxins was found, which is connected with generation of free radicals occurring in reactions of epoxidation, dechlorination, hydroxylation, formation of free oxygen, and stimulation of COX-2. Inflammatory reaction accompanying dioxin intoxication (syndrome chloracne) [11, 12] is the response to emission of large amount of free radicals and stimulation of COX-2. The result of chronic inflammatory reaction is occurrence of destructive changes in various organs connected with disordered metabolism of connective tissue [13, 14] and damage of cells.

The result of stimulation COX-2 is formation of proinflammatory thromboxanes and leukotrienes (HE and LXA4). The studies by Li and Matsumura [15] showed that TCDD, affecting AhR, initiates synthesis of proinflammatory interleukins as a result of activation of tract NF-*κ*B and within 1 hour it initiates a quick inflammatory reaction through synthesis of prostaglandins [15, 16].

The result of dioxin influence is increasing synthesis of CYP1A1, causes dioxin hydroxylation, and leads to formation of active oxygen species [15, 17, 18]. Increased secretion of free radicals contributes to destructive effect on collagen fibers occurring in blood vessels and being a scaffold for internal organs as well as causing lesions in bones [19, 20]. It contributes to occurrence of products of collagen degradation and destructive changes in microscopic observation of the organs affected by inflammatory process [13, 14, 21–23].

Premise for using TCP and ASA in latest literature reports revealed that both ASA and TCP, despite their properties such as blocking COX-2 and antioxidant activity, have an ability to block AhR [10, 18, 24–30].

The aim of the study was to determine the effect of dioxins on ultrastructure, cytological and histological structure of hippocampus—especially expression of estrogen receptors in CNS—and to define whether administration of TCP and ASA together and separately has a protective influence on the decrease of activity of proinflammatory effects in CNS.

## 2. Material and Methods

Experiments were performed on 36 female rats from the Buffalo strain, with body mass 140–160 g and aged 8–10 weeks. The animals stayed in air-conditioned rooms characterized by 15-cycle air exchange per 1 hour with temperature 22°C, humidity 55%, and light-day cycle 12/12 h. The study was approved by a local ethics council for animal experiments (permission number 38/2009).

Study groups of animals were made as a result of randomization and divided in the following way:

- group C is a control group of 6 females rats not exposed to effects of any agents, in which histological, ultrastructural, and immunohistochemical examinations were performed;
- group IP is a control group of 6 females rats, in which pleuritis was induced and then material for examinations was collected at 120 hours of pleuritis duration;
- group IPD is a TCDD treated group of 6 females rats, which 3 weeks before the examinations were administered with (i.m.) 5 *μ*g/kg b.w. of TCDD and in which after 3 weeks pleuritis was induced and then material for examinations was collected at 120 hours of pleuritis duration;
- group IPDE is a TCDD and TCP treated group of 6 females rats, which 3 weeks before the examination were administered with (i.m.) 5 *μ*g/kg b.w. of TCDD and every day for 3 weeks with *α*-tocopherol acetate at a dose of 30 mg/kg b.w. s.c. After 3 weeks pleuritis was induced and next material for examinations was collected at 120 hours of pleuritis duration;
- group IPDA is a TCDD and ASA treated group of 6 females rats, which 3 weeks before the examination were administered with (i.m.) 5 *μ*g/kg b.w. of TCDD and ASA at a dose of 50 mg/kg b.w. p.o. every day for 3 weeks. After 3 weeks pleuritis was induced and then material for examinations was collected at 120 hours of pleuritis duration;
- group IPDAE is a TCDD, TCP, and ASA treated group of 6 females rats, which 3 weeks before the examination were administered with (i.m.) 5 *μ*g/kg b.w. of TCDD and every day for 3 weeks *α*-tocopherol acetate s.c. at a dose of 30 mg/kg b.w. and ASA p.o. at a dose of 50 mg/kg b.w. were used. After 3 weeks pleuritis was induced and then material for examinations at 120 hours of pleuritis duration was collected.

In experiments TCDD (Sigma Aldrich, Poland) was used. It was dissolved in 1% solution of dimethyl sulphoxide (DMSO) in concentration 1 *μ*g/mL and administered intramuscularly into back limb muscles. In the studies *α*-tocopherol acetate was used in oily solution (Hasco-Lek, Poland) and it was used every day for 3 weeks at a dose of 30 mg/kg b.w. hypodermically (s.c.) in volume of 0.2 mL and ASA (Hasco-Lek, Poland) was administered orally in the form of suspension in starch solution at a dose of 50 mg/kg b.w. in volume of 0.5 mL every day for 3 weeks. Collection of material for examinations after 3 weeks since administration of TCDD was conditioned by obtained changes in organs in the previous research [31]. Induced pleuritis was caused by injection into pleural cavity between 5 and 6 right costal spaces of 1% carrageenin solution (Sigma Aldrich, Poland) in volume of 0.15 mL in a previously designed experimental model [13, 32].

At 120 hours of induced inflammatory reaction in particular groups of animals continuity of spinal cord was interrupted at height C1-C2 and then internal organs were collected and cranial vault was cut off and brain was dissected free—it was cut according to a schema included in stereotaxic atlas of rat brain [33]. Isolated rat brains from cranial cavity were prepared in order to visualize hippocampus structure from lateral cut 2.9–3.4 mm. Obtained from autopsy brain fragments, taking into account hippocampus structures, were placed in 4.0% buffered formalin solution assigned for histopathological and immunohistochemical examinations.

Preparations were fixed for 48 h, washed in running water, and dehydrated in series of alcohol from 50 to 100%. The obtained segments were scanned in methyl benzoate and xylene and paraffin-impregnated (POCH, Poland). After embedding selected segments in paraffin, paraffin blocks were cut on rotating microtome into sections 5 *μ*m thick and stained with hematoxylin and eosin according to Delafield (Sigma Aldrich, Poland) and Azan Novum according to Geidies (Süsse Labortechnik, Germany). Preparations were analyzed in optic microscope Nikon Eclipse, equipped with a program for morphometric analysis.

Immunohistochemical examinations were performed in order to determine estrogen receptors in hippocampus. Immunohistochemical determination of receptors was conducted with the use of tests LSAB + kit peroxidase (DAKO, Poland). Onto dewaxed and dehydrated tissue sections, after exposing epitopes by incubation in citrate buffer (Target Retrieval Solution, Citrate pH 6.0, DAKO, Poland) at temperature 97°C and blockage of endogenous activity of peroxidase by incubation with 3% H_2_O_2_ for 10 min, primary antibodies were put: monocline mouse antibody anti-estrogen *α* receptor MAB447 (Millipore, USA) diluted by buffer antibody diluents with background reducing components (DAKO, Poland) 1 : 800 and polyclonal rabbit antibody anti-estrogen receptor *β*-rabbit polyclonal IgG (Millipore, USA) 1 : 600. Incubation was carried out for 18 hours at temperature 4°C. Next, after rinsing sections in PBS (5 min) secondary biotinylated universal antibody IgG (Biotinylated Link IgG DAKO, Poland) was brought and incubated for 15 min at room temperature; then after washing sections in PBS (5 min) sections were incubated with a ready complex streptavidin-peroxidase (DAKO, Poland) for 15 min at room temperature. Next, DAB (3,3-diaminobezidinetetrachydrochloride, DAKO, Poland) was used at room temperature for 5 min. Additionally, sections were stained with hematoxylin (Sigma Aldrich, Poland). Final insoluble reaction product was obtained with different intensity of brown color. Next, sections were washed, dehydrated, and closed in Euparal (Paradox Co., Poland). Negative control of reaction specificity was conducted with omission of primary antibodies.

To confirm efficiency of immunohistochemical reaction positive control was conducted with the use of the same antibodies on hippocampus fragments of control female rats. Tests confirmed specificity of immunohistochemical reactions and immunoreactive estrogen receptors. Number of neurons was assessed per area unit in which reaction took place in nucleus and cytoplasm with reference to neurons not revealing reaction to antibodies.

Immunohistochemical preparations were analyzed in optic microscope Nikon Eclipse, equipped with a program for morphometric analysis.

For ultrastructural examinations in transmission electron microscope (TEM), prepared hippocampus fragments were fixed in 2.5% solution of glutaraldehyde (POCH, Poland) per 0.1 M phosphatic buffer with pH 7.4 for 4 hours. Next, the material was washed in the above mentioned phosphatic buffer and fixed for 2 h in 1.0% solution of osmium tetroxide (POCH, Poland) made on the basis of phosphatic buffer. Such prepared material was dehydrated in alcohol-acetone series (30–100%) and embedded in Epon 812 (Sigma Aldrich, Poland). Semithin sections (70 nm) were stained in 1.0% solution of toluidine blue (Millipore, USA) and ultrathin sections were contrasted with uranyl acetate (CHMES, Poland) and lead citrate (Sigma Aldrich, Poland). The material was analyzed in transmission electron microscope TEM EVO LS 15.

## 3. Results

### 3.1. Histopathological Assessment: Ammon's Horn

**Figure 1:**
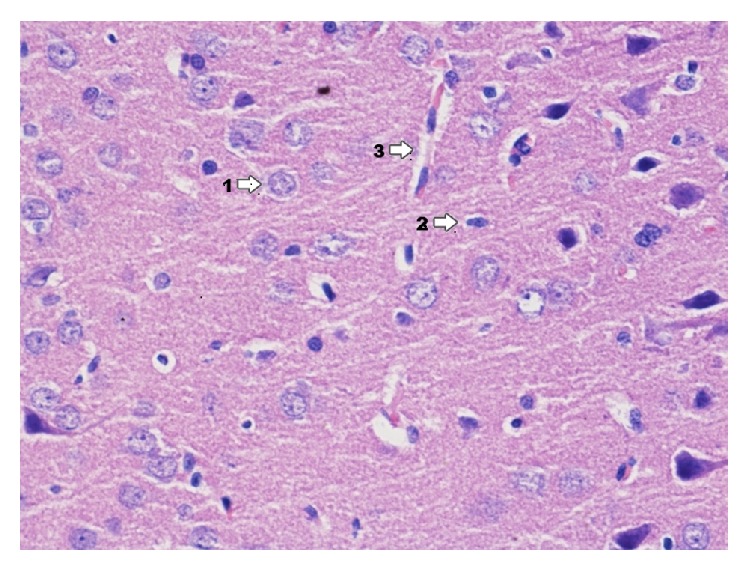
Histological image of hippocampus in animals in group C (enlargement 400x). In the image, numerous nerve cells (1), next to which there are glia cells (2), and astrocytes and oligodendrocytes, nearby which there are blood vessels (3).

Ammon's horn in control group C (Figure 1) is mainly made from pyramidal cells which form a diverse layer. Moreover there are nerve cells which form granular and molecular layer. In hippocampus of the control group pyramidal cells prevail as well as multilateral cells forming molecular layer.

**Figure 2:**
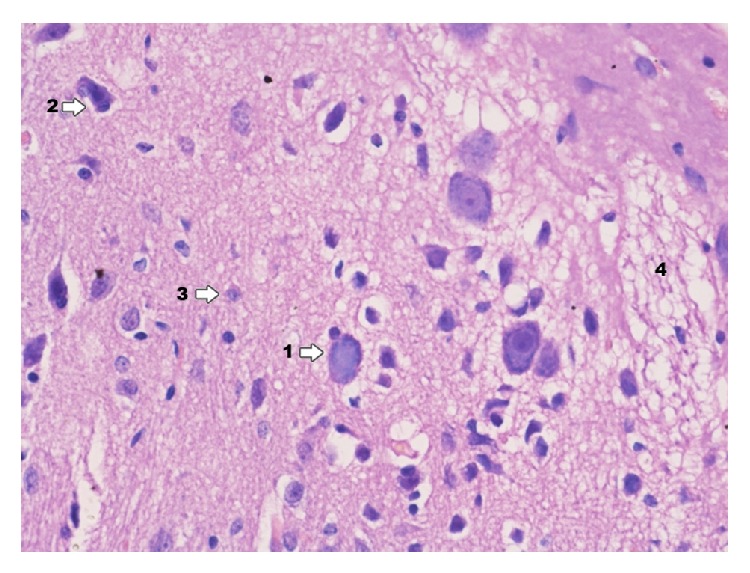
Histological image of hippocampus in animals in group IP (enlargement 400x). Nearby blood vessels, cells of microglia and macrophages agglomerate. Large multipolar neurons (1) are in the state of dispersion; between them there are smaller neurons (2) and cells of astroglia and oligodendroglia (3); nearby blood vessels there are microphages and macrophages present (micromesoglia cells); relaxation of weaving between neurons is noteworthy (4).

In the control group with induced inflammation IP (Figure 2) the clearest reaction concentrates around blood vessels. In those vessels one can notice slower blood flow, which is proved by retention of blood cells in the lumen of vessels in many places. Around blood vessels there is bigger concentration of microglia cells. Moreover glial proliferation was found. In those places dilutions of neuron weaving are visible and in some nerve cells in the area of perikaryon degeneration changes can be seen. Those changes mainly concern the area of granular nerve cells. To a smaller extent the above mentioned changes affect the layer of pyramidal cells. Vascular changes have a similar nature.

**Figure 3:**
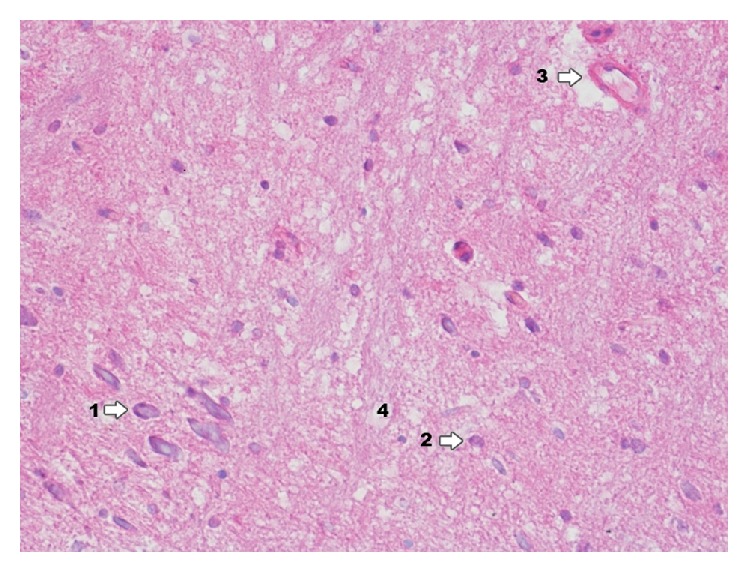
Histological image of hippocampus in animals in group IPD (enlargement 400x). Neurodegradation states of pyramidal neurons (1) and granule cells (2) are demonstrated; around blood vessels there is infiltration consisting of microglia cells and macrophages (3); in the area between nerve cells histological image presents foam structures (4).

In Ammon's horn in group IPD (Figure 3) there is high degree damage of grey matter of hippocampus; particularly clear degenerative changes can be seen in pyramidal cells and smaller ones in cells of granular and molecular layer. Delaminations between pyramidal cells and adjacent layers are confirmed. In particular pyramidal cells karyopyknosis of testicles can be seen as well as translucency of perikaryon cytoplasm. It also concerns the deviation zone of neuron processes. In a near zone (of analyzed neurons) proliferation of glia cells can be noticed. Degenerative changes in the above mentioned layers consist mainly in various degree of cytoplasmic vacuolation, which result in foam structure. Both in grey and in white zone there are changes displaying clamping of blood vessel lumen. In perivascular zones there is infiltration consisting of microglia cells and macrophages. Glial proliferation also applies to oligodendroglia cells, to a smaller extent to astroglia. A smaller degree of damage was found in molecular and granular layers.

**Figure 4:**
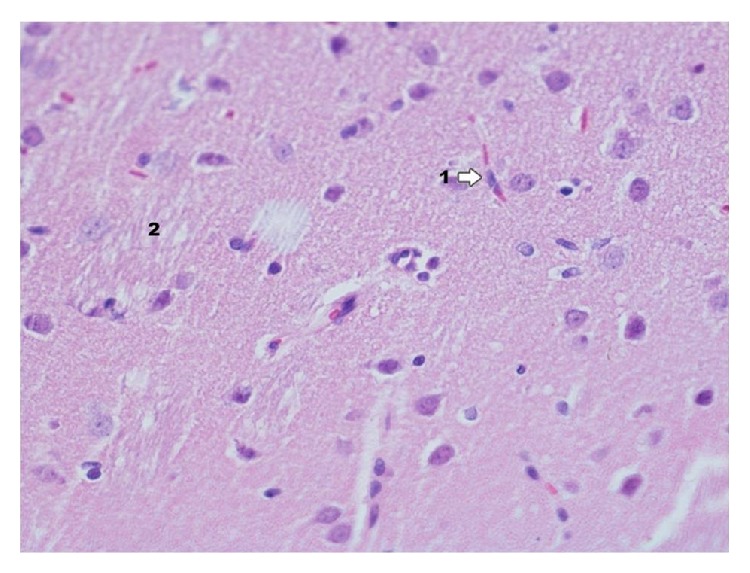
Histological image of hippocampus in animals in group IPDE (enlargement 400x). In the analyzed material the decrease of intensity of neurodegradation changes in neurons is observed; in the lumen of capillary vessels, there are numerous erythrocytes visible in rouleau formation (1), which may indicate the presence of venostasis; the foam structure between neurons is more poorly expressed (2).

In hippocampus in group IPDE (Figure 4) a smaller degree of damage of neurons occurs than in group IPD. The decrease of degenerative changes can be observed. It concerns all hippocampus zones of pyramidal cells as well as cells of granular and molecular layers. There is also a smaller degree of damage within blood vessels and glial proliferation is less intensified than in histopathological image in group IPD.

**Figure 5:**
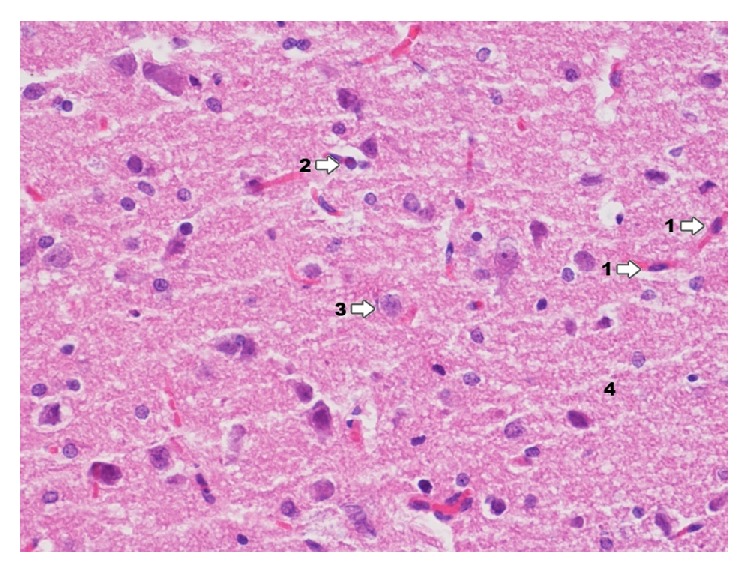
Histological image of hippocampus in animals in group IPDA (enlargement 400x). In the lumen of capillary vessels, there are numerous erythrocytes visible in rouleau formation (1), which may indicate the presence of venostasis; a small concentration of macrophages and microglia cells is observed in perivascular zone (2); in some ganglion cells, degradation states are found in the form of cytoplasmic vacuolation (3).

In Ammon's horn in group IPDA (Figure 5) damage of grey matter can be found to a smaller extent. Fundamental changes occur in pyramidal cells, to a smaller extent in molecular layer and cells of granular layer. Those changes are clearly emphasized near blood vessels and around them. In perikaryons of ganglionic cells cytoplasmic vacuolation is observed. It takes the form of foam structure. Granular cells also present foam structure,; however the scope of those changes is smaller than in pyramidal cells. Degenerative changes of nerve cells are accompanied by glial proliferation, especially of oligodendroglia and microglia cells. Microglia cells occur most clearly around blood vessels. A degree of damage of blood vessels is smaller than damage occurring in group IPD. Only in white matter the degree of damage of blood vessels stays at similar level like in animals, which did not receive salicylate.

**Figure 6:**
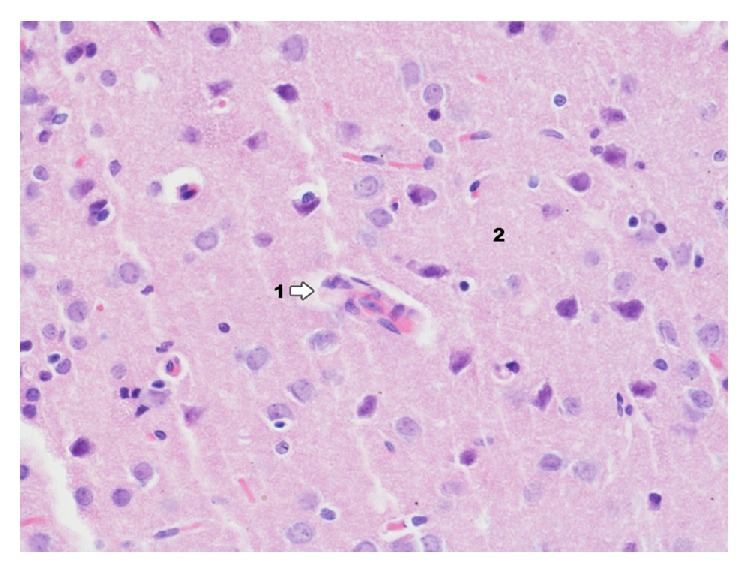
Histological image of hippocampus in animals in group IPDAE (enlargement 400x). Visible infiltration around blood vessels consisting of macrophages and microglia cells (1); sparse ganglion cells show neuron degradation states; however the whole image looks more favorable than in the previous experimental groups; spongy structure is more poorly expressed (2).

In Ammon's horn in group IPDAE (Figure 6) main changes concern the zone of pyramidal cells; to a smaller extent they apply to molecular layer and granular cells. The above mentioned changes most clearly are emphasized in direct and close vicinity of blood vessels. Cells of pyramidal layer reveal cytoplasmic vacuolation of perikaryon. In many cells of that layer, nuclei reveal karyopyknosis and beginnings of degenerative changes. Similar changes but of bigger intensity occur in granular cells. Granular cells in histological image present poorly expressed foam structure, which is the result of going on process of degeneration. In molecular layer there are smaller degree degenerative processes. Besides going on process of degeneration one can observe glial proliferation of microglia cells and oligodendroglia, which is particularly visible around blood vessels.

### 3.2. Histopathological Assessment: Dentate Gyrus

**Figure 7:**
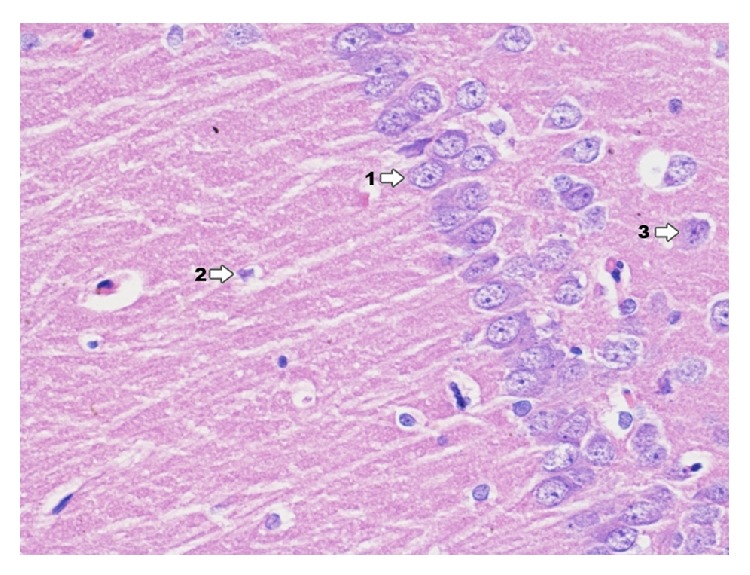
Histological image of dentate gyrus in animals in group C (enlargement 400x). In the image, visible numerous ganglion cells (1) forming into one layer, cells of molecular layer (2), and cells of granular layer (3) are shown.

In control group C (Figure 7) stroma for nerve cells is fibrous and protoplasmic astrocytes and oligodendrocytes. The structure of glia stroma is particularly evident in perivascular area (oligodendrocytes and protoplasmic astrocytes), whereas in a zone distant from vessels fibrous astrocytes prevail. The white matter zone consists of centripetal and centrifugal fibers, which are junctions between thalamus and hypothalamus. The structure of dentate gyrus presents grey matter arranged in 3 layers: molecular layer, granular layer, and layer of pleomorphic cells.

**Figure 8:**
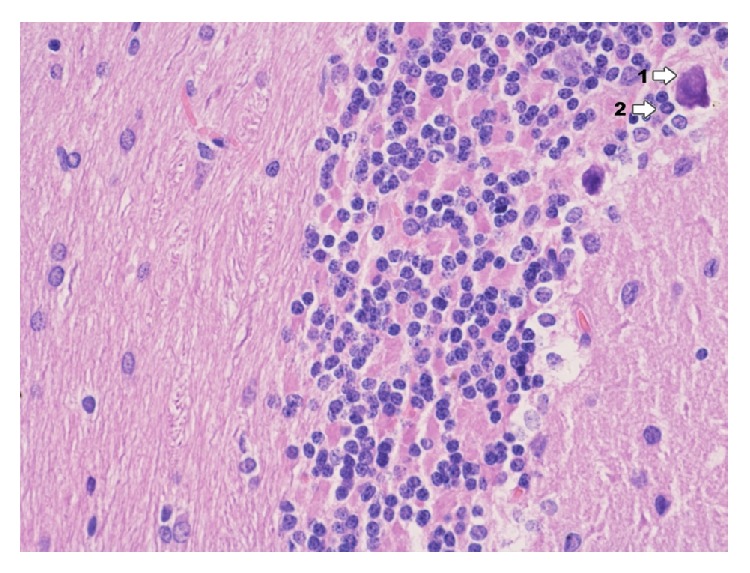
Histological image of dentate gyrus in animals in group IP (enlargement 400x). Basically correct image of structure with clear marking of the net of capillary blood vessels (1); in the lumen of capillary vessel numerous erythrocytes forming rouleau are shown, whose presence may indicate venostasis; near the above mentioned vessels there are macrophages and microglia cells present (2).

In control group with induced pleuritis IP (Figure 8) main changes occurring in the area of dentate gyrus concern granular and pyramidal layer. To a smaller extent they apply to molecular layer. The nature of changes is like in the area of Ammon's horn but the degree of those changes is bigger particularly in the layer of pyramidal cells. In the layer of granular cells a bigger glial proliferation than in Ammon's horn can be observed. In the lumen of capillary vessels rouleau formation of erythrocytes is found which may prove hemostasis in blood vessels. Most clear changes of degenerative nature were observed in molecular layer.

**Figure 9:**
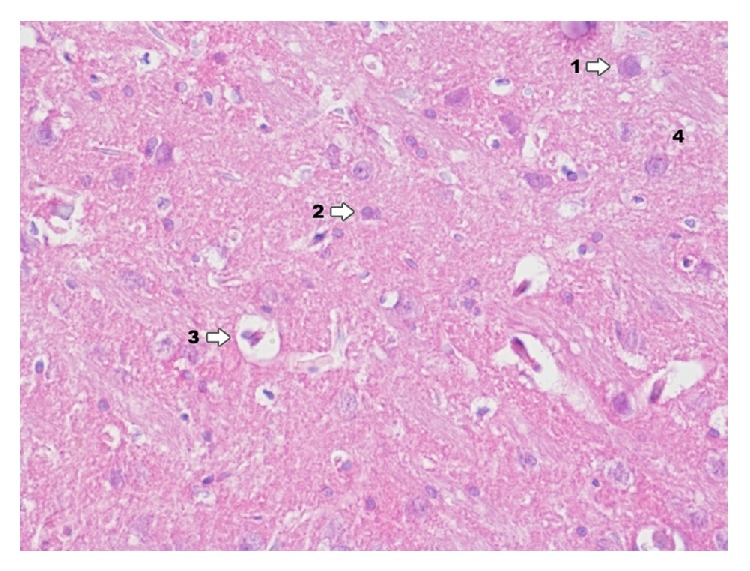
Histological image of dentate gyrus in animals in group IDP (enlargement 400x). It reveals neuron degradation states of pyramidal neurons (1) and granular cells (2); around blood vessels there is infiltration consisting of microglia cells and macrophages (3); in the area between nerve cells histological image presents foam structures (4); image of foam structure is more poorly expressed.

In dentate gyrus in group IPD (Figure 9) degenerative changes of neurons are more poorly expressed, they concern pyramidal neurons and granular cells, and glial proliferation occurs in the perivascular zone. Around blood vessels there is infiltration consisting of microglia cells and macrophages.

**Figure 10:**
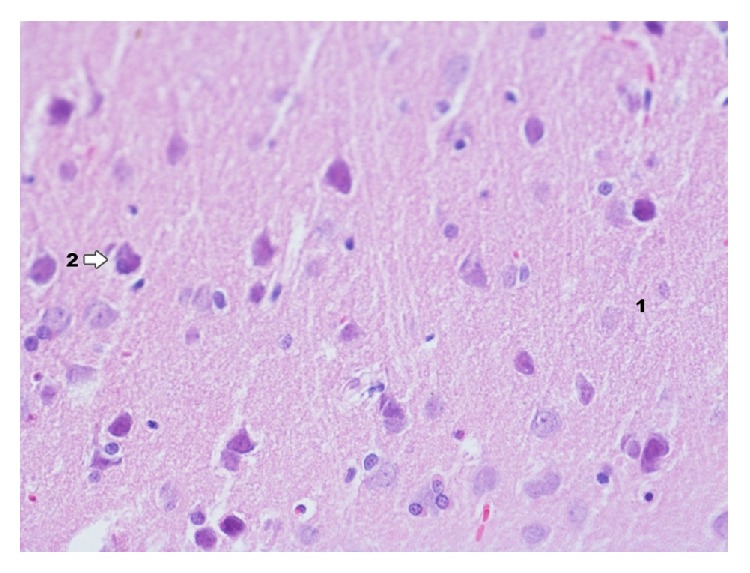
Histological image of dentate gyrus in animals in group IDPE (enlargement 400x). Histological image of dentate gyrus is similar to hippocampus in this group.

Histopathological assessment of dentate gyrus in group IPDE (Figure 10) is similar to histopathological image of dentate gyrus in group IPDAE. It covers more poorly expressed degenerative changes of nerve cells in the area of dentate gyrus and glial proliferation occurs mainly in the blood vessel zone, where infiltration of microglia cells is observed.

**Figure 11:**
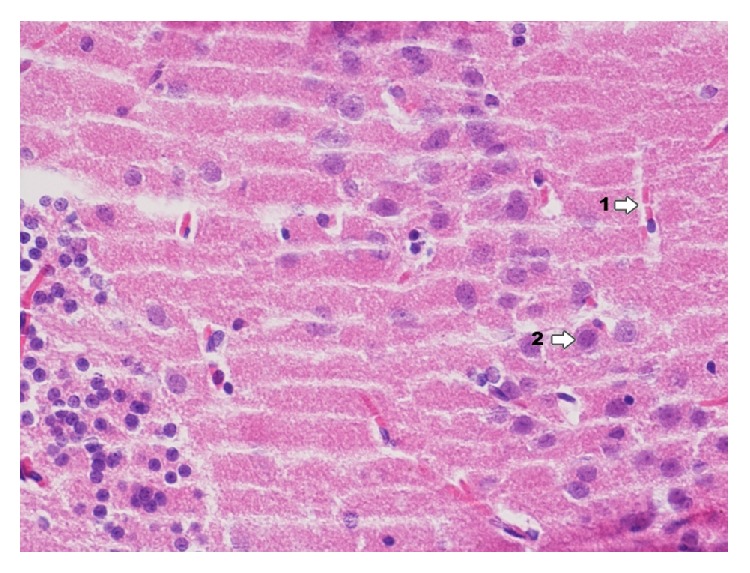
Histological image of dentate gyrus in animals in group IDPA (enlargement 400x). In the lumen of capillary vessels, there are numerous erythrocytes visible in rouleau formation (1), which may indicate the presence of venostasis; neurodegradation states of neurons (2), which are more poorly expressed than in Ammon's horn.

**Figure 12:**
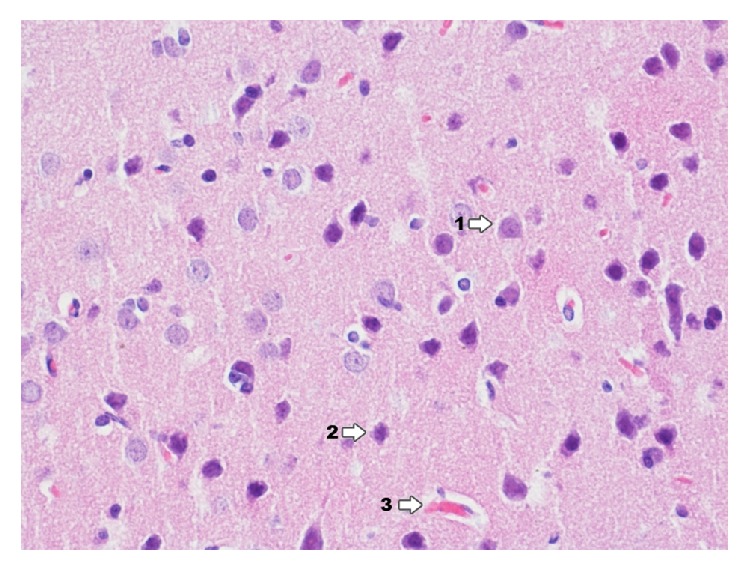
Histological image of dentate gyrus in animals in group IDPAE (enlargement 400x). Neuron degradation states occur on a similar level like in Ammon's horn and mainly concern pyramidal cells (1), whereas granular cells (2) show more advanced degenerative processes of neurons; in the lumen of capillary vessels, there are numerous erythrocytes visible in rouleau formation (1), which may indicate the presence of venostasis.

In group IPDA (Figure 11) in the area of dentate gyrus there are changes like in Ammon's horn but the degree of changes is smaller. Degenerative changes occur focally. In dentate gyrus in group IPDAE (Figure 12) the nature of changes is like in Ammon's horn but the degree of intensity is smaller. Degenerative changes occur focally and concentrate mainly in pyramidal cells and the granular layer cells.

### 3.3. Immunohistochemical Reaction to the Presence of Estrogen Receptors *α* (ER*α*): Ammon's Horn and Dentate Gyrus

**Figure 13:**
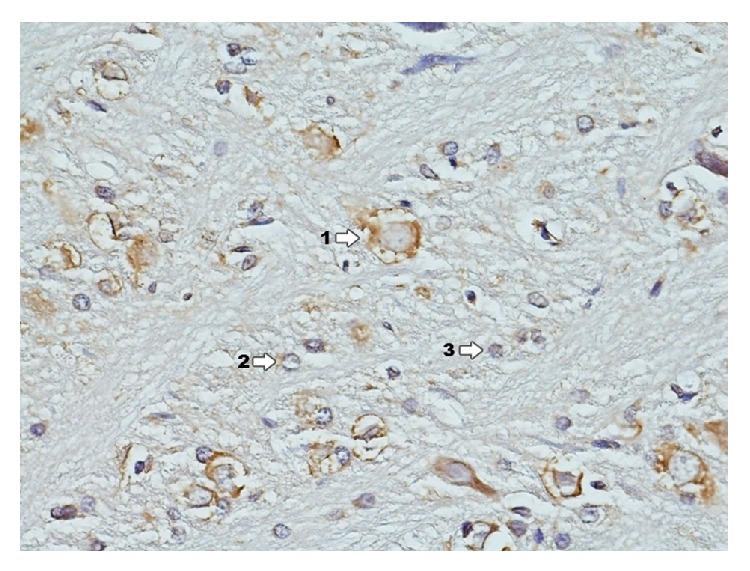
Immunohistochemical reaction towards ER*α* in the structures of hippocampus and dentate gyrus in group C (enlargement 400x). Clear expression of ER*α* concentrates in large multipolar nerve cells—ganglia (1); a smaller degree of expression is in granular cells (2) whereas there is lack of it in glia cells (3); border between white and grey matter.

Conducted immunohistochemical analysis for the presence of ER*α* and their expression in structures of Ammon's horn in control group (Figure 13) revealed expression of estrogen receptors in large pyramidal cells, whereas smaller degree of expression was observed in granular cells. There was lack of reaction in glia cells. In structures of dentate gyrus cells of granular layer reveal expression.

**Figure 14:**
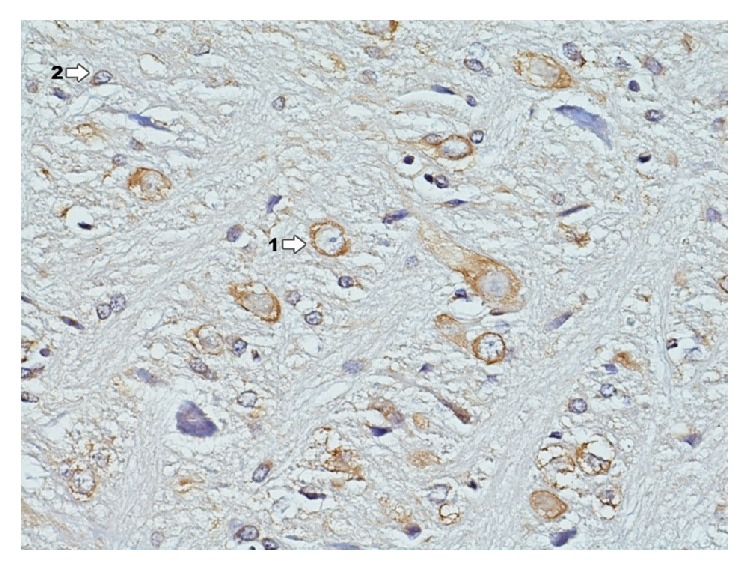
Immunohistochemical reaction towards ER*α* in the structures of hippocampus and dentate gyrus in group IP (enlargement 400x). Clear expression of ER*α*, especially in pyramidal ganglion cells (1) and to a smaller extent in granular cells (2).

In control group with induced pleuritis IP (Figure 14) the clearest immunohistochemical reaction to the presence of ER*α* occurs in the zone of granular cell layer. Reaction concentrates in perikaryons around nucleus. In the zone of neurons located near blood vessels the reaction is more intense. In the area of proliferating glia cells there is a clear decrease of concentration of immunohistochemical reaction. Immunohistochemical reaction to the presence of ER*α* in the area of dentate gyrus is significantly weaker and concerns cells of granular and molecular layers. No reaction is found in pyramidal cells, which undergo neurodegradation.

**Figure 15:**
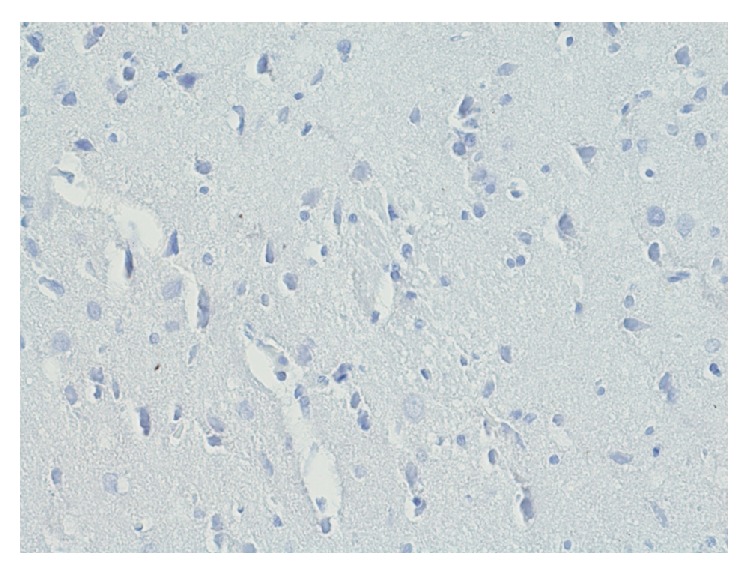
Immunohistochemical reaction towards ER*α* in the structures of hippocampus and dentate gyrus in group IPD (enlargement 400x). There is complete lack of immunohistochemical reaction to ER*α* in hippocampus and dentate gyrus.

In the group exposed to dioxin effect with induced inflammation of IPD (Figure 15) in the area of Ammon's horn and dentate gyrus, immunohistochemical reaction to the presence of ER*α* does not occur. No expression of estrogen receptors is found in this area.

**Figure 16:**
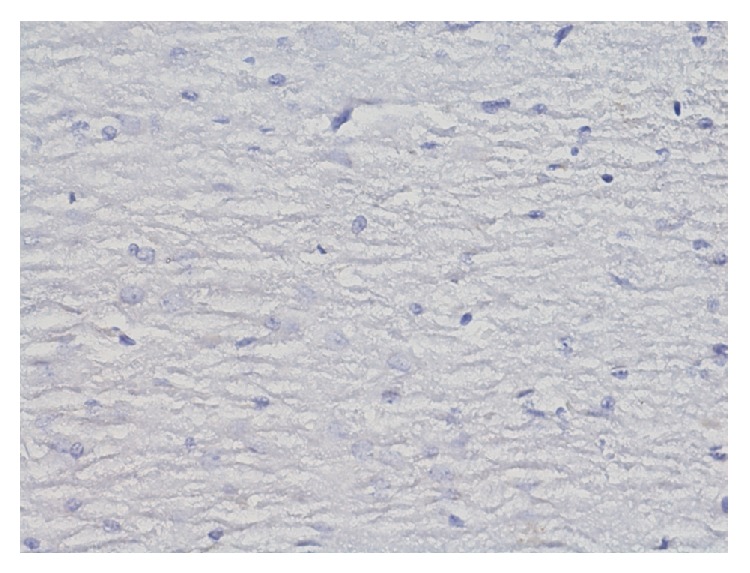
Immunohistochemical reaction towards ER*α* in the structures of hippocampus and dentate gyrus in group IPDE (enlargement 400x). No expression of ER*α*.

In the area of hippocampus and dentate gyrus in group IPDE (Figure 16) immunocytochemical reaction to the presence of ER*α* does not occur. No expression of estrogen receptors is found in that area.

**Figure 17:**
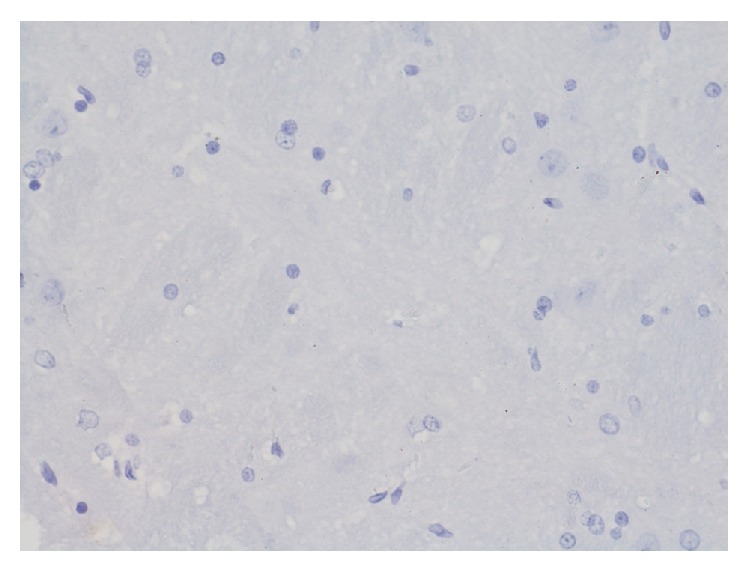
Immunohistochemical reaction towards ER*α* in the structures of hippocampus and dentate gyrus in group IPDA (enlargement 400x). No expression of ER*α*.

In group IPDA (Figure 17) in the area of Ammon's horn immunocytochemical reaction to the presence of ER*α* does not occur. There is no expression of estrogen receptors.

**Figure 18:**
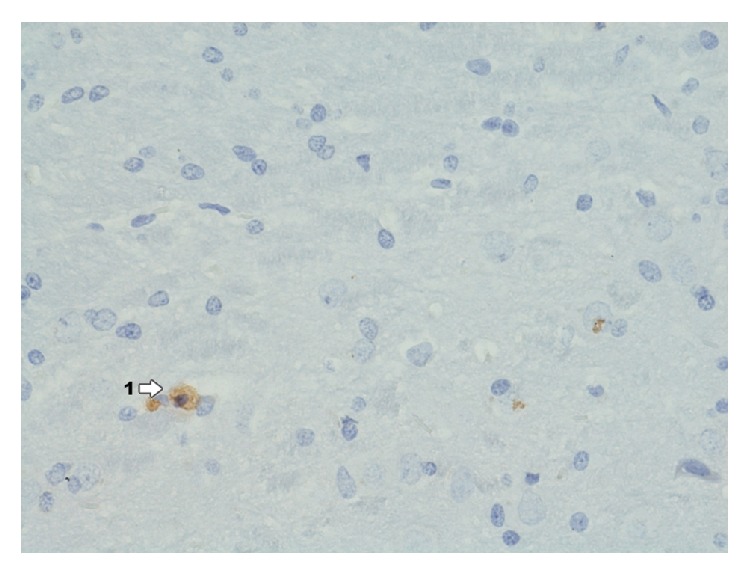
Immunohistochemical reaction towards ER*α* in the structures of hippocampus and dentate gyrus in group IPDAE (enlargement 400x). In Ammon's horn and dentate gyrus, amounts of immunohistochemical reaction (1) are traced.

In Ammon's horn in group IPDAE (Figure 18) immunohistochemical reaction to the presence of ER*α* is much worse determined than in group IP. In damaged pyramidal cells reaction is hardly observed. In those cells, in advanced state of degeneration (foam structure of cytoplasm) reaction is completely absent. In cells of granular layer reaction is also poorly determined. Most cells of blood vessel endothelium reveal damage (edema). In the group of animals exposed to dioxin action and additionally administered with TCP and ASA, evident reduction of immunohistochemical reaction of neurons was found compared to group IP, whereas smaller proliferation of glia cells and smaller changes in blood vessels were observed compared to those in group IPD.

### 3.4. Ultrastructural Assessment of Hippocampus Structures

**Figure 19:**
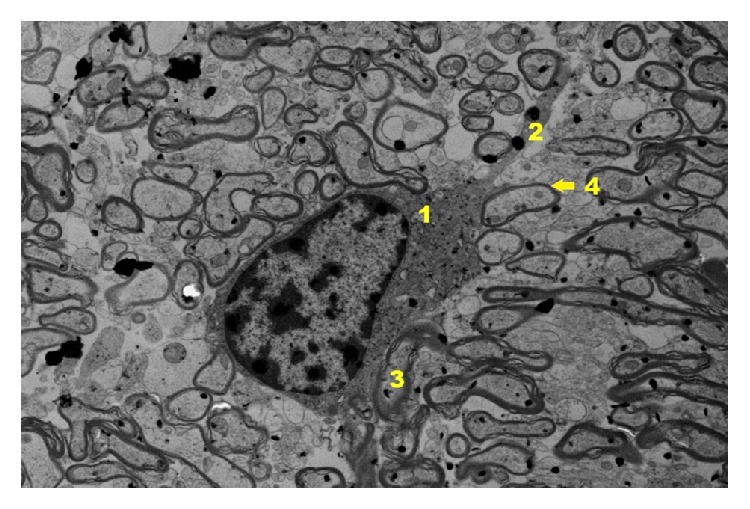
Ultrastructural image of neurofibrils of hippocampus in group C (enlargement 5000x). In the image, nerve cell from pyramidal zone (1); nerve cell process (2); axoplasm (3); myelin sheath (4) are shown.

Ultrastructural images of control group (Figure 19) present clearly shaped myelinated fibers. Cells of pyramidal layer reveal a correct ultrastructural image. In nucleus euchromatin prevails whereas heterochromatin concentrates in the form of insulas and bands mainly near nuclear areola. In cytoplasm granular endoplasmic reticulum prevails as well as other cell organelle engaged in the process of neurotransmission. Myelinated fibers are surrounded by myelin sheath. In axoplasm elements of reticulum and relatively numerous mitochondria can be observed.

**Figure 20:**
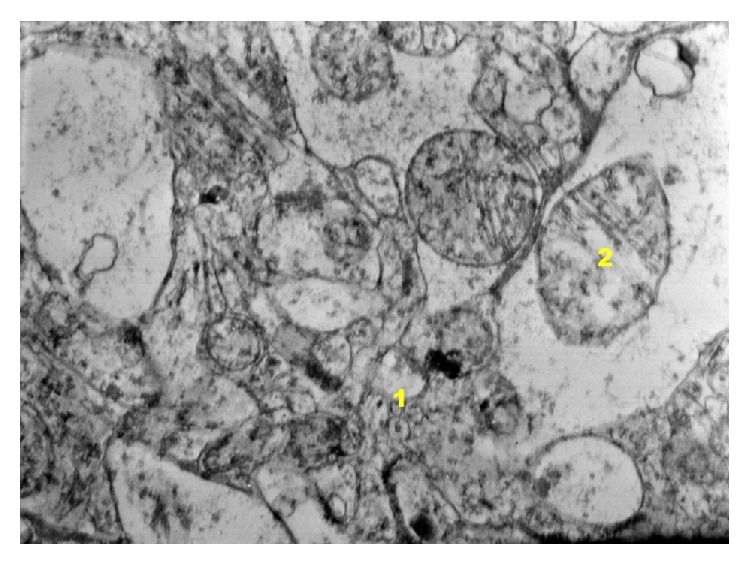
Ultrastructural image of neurofibrils of hippocampus in group IP (enlargement 32 000x). The state of neuron degeneration has been found as well as vacuolation of endoplasmic reticulum (1) and mitochondria edema (2).

In control group with induced inflammation of IP (Figure 20) in neuron perikaryon there are mitochondria being in different state of damage, while their edema prevails. Moreover mitochondria with correct structure are observed. Mitochondria with occurring edema are 30% of their population. The other part of perikaryon cytoplasm reveals basically correct structure; however areas of clarifications within the zone of endoplasmic reticulum can be found. The zone of granular reticulum mostly presents a correct image but in some places ectasias of rough endoplasmic reticulum can be observed. The above mentioned image is also confirmed in the area of axoplasm. Glia cells are unchanged. Dilation of space in nuclear areola is found. Significant changes in nerve fibers were not found. In some cells of oligodendroglia degenerative changes occur which consist in vacuolation of endoplasmic reticulum. In the area of capillary blood vessels vacuolation of endothelium cells can be found in some places as well as mitochondria edema. Macrophages are often visible in the zone adjacent to adventitia of capillary vessels. Endothelium cells of the above mentioned vessels are softened; in some of them the process of degeneration can be observed. Oligodendrocytes occurring near blood vessels do not reveal bigger changes in their structure. Some oligodendroglia cells adhering to blood vessels show symptoms of softening and some undergo degeneration.

**Figure 21:**
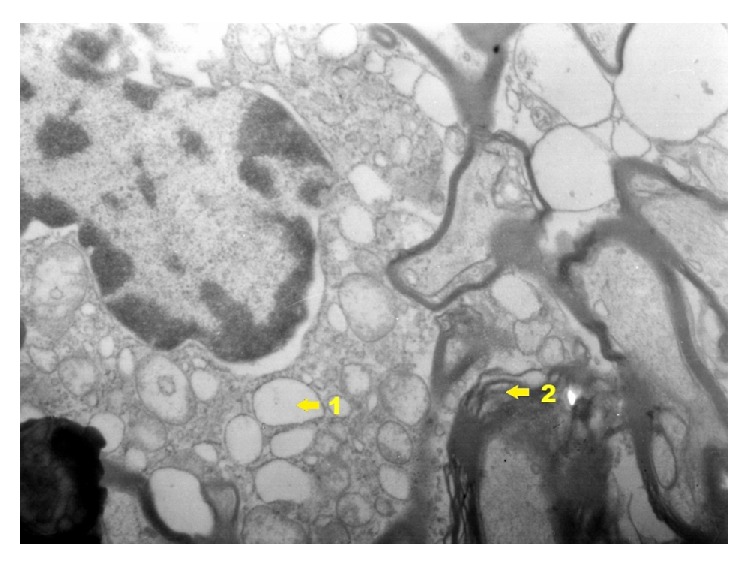
Ultrastructural image of neurofibrils of hippocampus in group IPD (enlargement 18 000x). There is a clear degree of vacuolation of neuron cytoplasm (1); nerve fibers situated near nerve cell reveal the image of delamination of myelin sheath laminae (2).

In the group exposed to dioxin action with induced inflammation of IPD (Figure 21) occurrence of significant changes in ultrastructure of hippocampus. In perikaryon of nerve cells dilation of perinuclear space was stated. In the area of nucleus heterochromatin concentrates in the form of insulas or bands by nuclear membrane. Clearly exposed nucleolus with areas indicated that its disintegration was localized with vesicular ectasia of endoplasmic reticulum and clarifications of basic cytoplasm. Moreover mitochondria edema and dispersion of structures of Golgi apparatus were observed. Present myelinated fibers adjacent to the described perikaryon sometimes reveal vacuolation of axoplasm. In axoplasm there is also vacuolation of endoplasmic reticulum and mitochondria edema. In the process zone, segmentary delamination of myelin sheaths particularly myelinated fibers is found. In glia cells the following things are visible: vacuolation of cell cytoplasm and mitochondria edema, dispersion of reticulum within axoplasm and glia cells, and delamination of neurofilaments. Cells of glia tissue especially oligodendrocytes reveal degenerative changes of similar intensity like in nerve cells. In astroglia cells a smaller degree of damage can be found. The character of changes in oligodendrocytes takes form of vacuolation of endoplasmic reticulum and basal cytoplasm. General image of changes presents the state of cell edema. In blood capillary vessels there are delaminations between endothelium and adventitia, softening of endothelium cells, mitochondria edema, degenerative changes, and cell vacuolation. Moreover increased fibrogenesis can be observed and at times occurring connective tissue leads to clamping of capillary vessel lumen. In vessels there are numerous erythrocytes squeezing through their lumen and near vessel there are macrophages.

**Figure 22:**
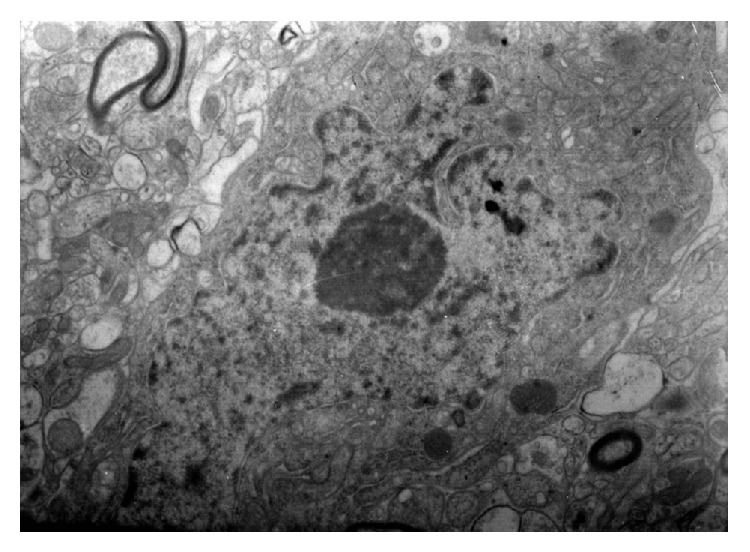
Ultrastructural image of neurofibrils of hippocampus in group IPDE (enlargement 32 000x). Few visible images of mitochondria edema; the image of structure is basically correct.

Insignificant vacuolation was noticed in the zone of axoplasm in group IPDE (Figure 22). In glia cells of that group mitochondria edema is found. Glia cell remains basically correct. The perinuclear space has a stable form; appeasement of degenerative process is observed in nerve cells. Blood vessels have a correct structure. Endothelium cells preserve a correct image; there are no degenerative changes in oligodendroglia cells.

**Figure 23:**
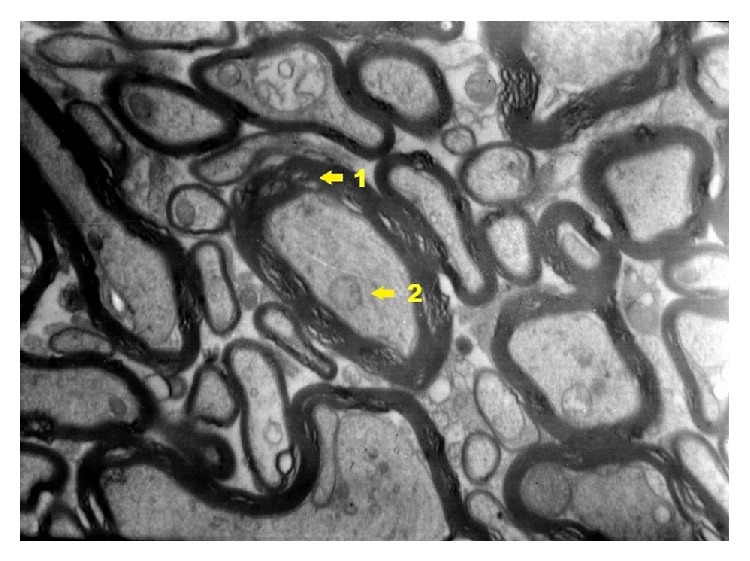
Ultrastructural image of neurofibrils of hippocampus in group IPDA (enlargement 18 000x). In some nerve fibers segmental delamination of myelin sheath laminae can be noticed (1); in axoplasm of analyzed nerve fibers there are mitochondria edema and vacuolation of endoplasmic reticulum (2).

In group IPDA (Figure 23) a smaller degree of damage of neurons is found. Proliferation of glia tissue is maintained on similar level like in group IPDE. Ectasia of cisterns of endoplasmic reticulum is visualized. Damage of blood vessels is also smaller. In some nerve cells damage of myelin sheaths is maintained. Stabilization of majority of cell structures takes place. Endothelium of blood vessels presents the correct image.

**Figure 24:**
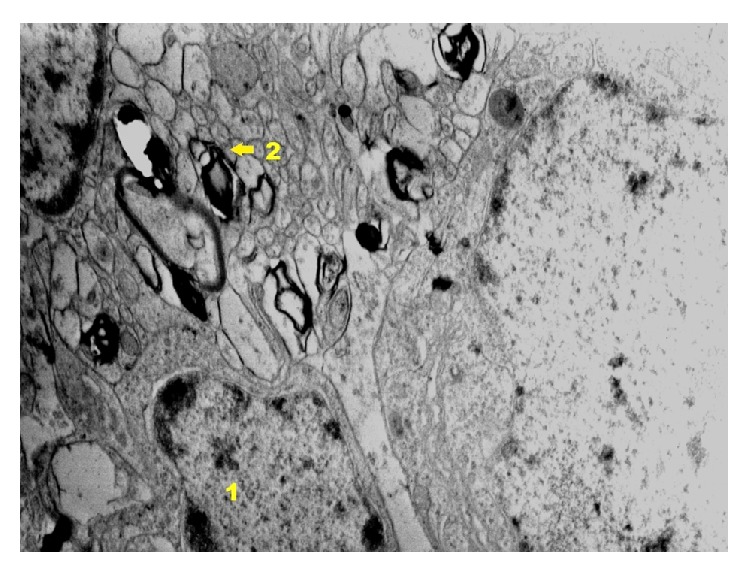
Ultrastructural image of neurofibrils of hippocampus in group IPDAE (enlargement 24 000x). States of axoplasm vacuolation are present. Structural image of oligodendroglia cells reveals the correct image (1); nerve fibers situated near nerve cells show a smaller degree of delamination of myelin sheath laminae (2); nerve cells reveal basically correct state; in some places dilation of endoplasmic reticulum can be seen.

In cytoplasm of nerve cells in group IPDAE (Figure 24) vacuolation, which takes focal character, was stated. Mitochondria edema occurs. Delaminations of myelin sheaths are not observed. The adaptive state in glia cells is shown. Stabilization of cell structures occurs, especially in the area of endoplasmic reticulum. Endothelium cells are unchanged. Even bigger weakening of neuron reaction is observed as well as very small proliferation of glia cells and sporadically occurring changes in blood vessels. Typical vessels for the inflammation, softening of endothelium cells and basement membrane, larger vacuolation of oligodendrocytes near vessels, and present macrophages were exposed.

## 4. Discussion

This study has showed that estrogens affect density of dendritic endings in the field CA1 of hippocampus. With decreased concentration of estrogens there is correlated decrease of density of those endings. Moreover it has been revealed that estrogens have an impact on formation of endings between neurons, affecting neuron functions, which was proved by previous studies. It was found that estrogens are also formed within the area of brain in neurons and astrocytes from androgens under the influence of aromatizing enzyme (P-450 AROM and CYP19). It should be presumed that a reason for disturbances in the estrogen synthesis may be dioxins, which through AhR intensify the CYP synthesis and P-450 synthesis. That can be confirmed by numerous conclusions resulting from other studies [15, 16, 18].

In our own studies assessing expression of estrogen receptors ER*α* in Ammon's horn and dentate gyrus in the group of animals exposed to dioxin action (IPD) positive immunohistochemical reaction to the presence of ER*α* in the mentioned brain areas was found. The obtained results which stated lack of estrogen receptors in the areas of hippocampus and dentate gyrus in animals exposed to TCDD action are confirmed by the other research [11, 34–42]. A hypothesis concerning disorders of CNS functions as a result of generation of free radicals and TNF as a result of TCDD effects [1, 43] finds its confirmation in histopathological and ultrastructural image of Ammon's horn and dentate gyrus. Degenerative changes of pyramidal cell of hippocampus were found as well as perivascular inflammatory states, which are signs of inflammatory infiltrations (microglia cells and macrophages). Ultrastructural image indicates significant metabolism disorders in nerve cells, which is proved by vacuolation of endoplasmic reticulum and mitochondria edema as well as disordered neurotransmission, which is connected with delamination of myelin sheath laminas.

Destructive changes in nerve cells and disorders of mitochondria functions are associated with increased oxidative stress, in which proinflammatory TCDD action overlaps induced inflammatory changes, which is proved by other studies [44–46]. Inflammatory changes in capillary vessels of hippocampus are also manifested in delamination between endothelium, adventitia, and increased fibrogenesis. Described phenomenon associated with occurrence of apoptotic features at 120 hours of inflammatory reaction in an organ distant from the organ with inflammatory process (cardiac muscle, e.g.) [47] can be also confirmed in the results from this study. In the group of animals with control inflammation at 120 hours IP larger concentration of microglia cells and glial proliferation in hippocampus was revealed as well as dilution of neuron weaving and degenerative changes in the area of perikaryon compared to the image of hippocampus in control group IP. Larger concentration of microglia cells occurs around blood vessels. In ultrastructural image mitochondria edema can be found as well as correct mitochondria and unchanged substructure of neuron cytoplasm. In the perivascular area vacuolation and edema of endothelium cells can be found in some places.

Inflammatory reaction in histopathological image of hippocampus and other organs on the basis of our own studies and other authors' [10, 47–50] may proceed in a few stages extended in time. It was found that in an initial period the change of permeability of blood vessels takes place and water with little protein fractions gets into intercellular space and in the later period proteins with bigger relative molecular mass appear and cause edema, hypoxia, and ectasia of capillary vessels. Due to effects of various mediators and proinflammatory cytokines, destruction of vascular endothelia follows and the result is exposing collagen fibers of blood vessels [51]. The consequence of that is platelet aggregation connected with occurrence of microclots, thus leading to hemodynamic changes, which also result from changes in blood composition such as increase of blood viscosity, rouleau formation of erythrocytes, and ability of their hemolysis.

Lauritzen et al. [50] observed the decrease of concentration of vitamin E in blood serum during pneumonia, which can be the cause of increased permeability of lysosome membrane for enzymes which may result in damage of cell structure, mitochondria membranes, and endoplasmic reticulum. Inflammatory reaction increased by TCDD intensifies the use of endogenous TCP [52] and as a result the increase of free radicals stimulating enlarged collagen synthesis which effects were observed in our own studies among animals exposed to TCDD action [10].

Immunohistochemical assessment of Ammon's horn and dentate gyrus in the group of animals exposed to action of dioxin and TCP (IPDE) with regard to expression of estrogen receptors ER*α* revealed lack of positive immunohistochemical reaction to the presence of ER*α* in the mentioned brain areas. Accordingly, it is considered that TCP and its mechanism of action do not stimulate of estrogen receptor, which is blocked by TCDD in hippocampus and dentate gyrus.

Histopathological assessment of Ammon's horn and dentate gyrus revealed reduction of degenerative changes in pyramidal cells and cells of granular and molecular layers as well as smaller glial proliferation in the blood vessel zone. In the assessment of ultrastructure of Ammon's horn and dentate gyrus sparse mitochondria edema can be found in oligodendroglia cells. In nerve fiber axoplasm of hippocampus slightly advanced states of vacuolation, edema of endoplasmic reticulum, and mitochondria are visible. Areas of blood vessels and endothelium do not reveal degenerative changes associated with inflammatory process.

On the basis of histopathological and ultrastructural hippocampus of animals exposed to TCDD action and TCP one can state protective TCP effect which reduces the observed inflammatory reactivity in the group of females IPD. Using TCP in animals intoxicated with dioxins limits changes in histopathological and ultrastructural image. Analysis of immunohistochemical reaction towards the presence of estrogen receptors in areas of dentate gyrus and hippocampus among animals exposed to TCDD action, administered with ASA and with induced inflammatory reaction, revealed lack of expression of estrogen receptors in hippocampus; however their trace presence was found in dentate gyrus. The obtained result suggests that using ASA partly abolishes negative effects of TCDD action on expression of ER*α*. Effects of TCDD action, consisting in stimulation of COX-2 and combining with AhR, are eliminated by using ASA. Thus proinflammatory effects of dioxin action are eliminated. However it is hard to explain occurrence of trace expression of ER*α* after using ASA in animals exposed to TCDD action, which does not occur among animals in group IPD.

In histopathological image of hippocampus in group IPDA smaller damage of grey matter concerning pyramidal cells was found and to a smaller extent—molecular and granular layer as well as vacuolation of perikaryon cytoplasm of ganglionic and granular cells and to a smaller extent—damage of blood vessels with microglia infiltrations compared to group IPD, whereas in dentate gyrus degenerative changes occur focally and a degree of neuron damage is smaller than in hippocampus. The image of ultrastructure of hippocampus of animals exposed to effects of TCDD and ASA reveals a smaller degree of damage of nerve cells and blood vessels, in which endothelium does not reveal any changes. Proliferation of glia tissue can be seen to the same degree as in group IPDE. In some nerve cells the state of damage of myelin areolas remains. Stabilization of most cell structures takes place.

Histopathological and ultrastructure assessment of hippocampus of animals exposed to TCDD action, in which for 3 weeks ASA was used, reveals a significant improvement compared to group IPD and some similarities to group IPDE. It also proves that ASA eliminates proinflammatory effects of dioxin action connected with damage of cell structures of hippocampus and dentate gyrus. The obtained results can be explained by observations made by Maharaj et al. [28], who showed that ASA inhibits production of superoxide anions, formation of products of lipid peroxidation, and reduction of PGE2 and TXB2 concentration connected with inflammatory reaction, thus protecting hippocampus against neurodegenerative changes [24, 48, 53].

It is interesting to find occurrence of clear but small in quantity positive reactions to the presence of estrogen receptor in group IPDAE. This fact opts for protective action of those substances administered together against destructive effects of TCDD on expression of ER*α*. Histopathological assessment of Ammon's horn and dentate gyrus showed that granular cells and molecular layer cells have poorly expressed foam structure, which is the result of proceeding degeneration. The observed changes in perivascular area are characterized by glial proliferation with weaker intensity than in group IPD. Infiltrations around blood vessels consisting of macrophages and microglia cells are less intensified than in group IPD.

Analysis of ultrastructure of Ammon's horn revealed that stabilization of cell structures occurs particularly in the area of endoplasmic reticulum and delaminations of myelin sheath laminas are not observed. Vacuolation of neuron cytoplasm takes the focal character; mitochondria edema occurs. Occasionally there are changes in blood vessels associated with softening of endothelium cells and basement membrane, larger vacuolation of oligodendrocytes near vessels, and the presence of macrophages (ultrastructural image opts for existing local inflammatory changes).

The above histopathological and ultrastructural image indicates that in group IPDAE degenerative changes of proliferation of perivascular infiltrations and softening of vascular endothelium occur with much smaller intensity than in group IPD. A part of changes observed in group IPD, opting for a significant damage of hippocampus and associated with degeneration of pyramidal cells, granular cells, delamination of myelin sheaths, and vacuolation of cytoplasm, does not occur in group IPDAE, which proves protective action of TCP and ASA used simultaneously and for a longer time since the moment of intoxication with dioxin. Using TCP and ASA separately does not reveal such significant action protecting CNS against destructive and inflammatory changes which are the results of dioxin effects.

## 5. Conclusion

Dioxin contributes to atrophy of estrogen receptors in hippocampus, in which also destructive and inflammatory changes were found along with demyelination of myelin sheaths. Histopathological and ultrastructural assessment of hippocampus and dentate gyrus showed the decrease of destructive changes and weakening of inflammatory reaction in this area and it indicated reduction of both degenerative and inflammatory changes in the area of blood vessels which opts for protective action of ASA towards CNS structures. Moreover total protective action of TCP and ASA towards CNS functions was stated. Histopathological and ultrastructural image of hippocampus areas in rats, in which both TCP and ASA were used, is characterized by poorly expressed degenerative changes and smaller inflammatory reactivity. Poorly expressed immunohistochemical reaction to the presence of ER*α* occurs. The conducted studies authorize stating that combined administration of TCP and ASA in animals exposed to TCDD effects reveals bigger protective action than administration of those substances separately.

## References

[1] Phillis J. W., Horrocks L. A., Farooqui A. A. (2006). Cyclooxygenases, lipoxygenases, and epoxygenases in CNS: their role and involvement in neurological disorders. *Brain Research Reviews*.

[2] Ishida T., Matsumoto Y., Takeda T., Koga T., Ishii Y., Yamada H. (2010). Distribution of 14C-2,3,7,8-tetrachlorodibenzo-p-dioxin to the brain and peripheral tissues of fetal rats and its comparison with adults. *Journal of Toxicological Sciences*.

[3] Teraoka H., Kubota A., Dong W. (2009). Role of the cyclooxygenase 2-thromboxane pathway in 2,3,7,8-tetrachlorodibenzo-p-dioxin-induced decrease in mesencephalic vein blood flow in the zebrafish embryo. *Toxicology and Applied Pharmacology*.

[4] Byers J. P., Masters K., Sarver J. G., Hassoun E. A. (2006). Association between the levels of biogenic amines and superoxide anion production in brain regions of rats after subchronic exposure to TCDD. *Toxicology*.

[5] Akahoshi E., Yoshimura S., Ishihara-Sugano M. (2006). Over-expression of AhR (aryl hydrocarbon receptor) induces neural differentiation of Neuro2a cells: Neurotoxicology Study. *Environmental Health*.

[6] Wang X., Hawkins B. T., Miller D. S. (2011). Aryl hydrocarbon receptor-mediated up-regulation of ATP-driven xenobiotic efflux transporters at the blood-brain barrier. *The FASEB Journal*.

[7] Piskorska-Pliszczyńska J. (1996). The toxicity and dioxins mode of action. *Veterinary Medicine*.

[8] Poland A., Teitelbaum P., Glover E. (1989). [125I]2-iodo-3,7,8-trichlorodibenzo-p-dioxin-binding species in mouse liver induced by agonists for the Ah receptor: characterization and identification. *Molecular Pharmacology*.

[9] Madej J. A. (2004). Estrogens in cancer of reproductive organs. *Veterinary Medicine*.

[10] Całkosiński I., Rosińczuk-Tonderys J., Szopa M., Dobrzyński M., Gamian A. (2011). High doses of tocopherol in the prevention and potentiation of dioxin in experimental inflammation-potential application. *Advances in Hygiene and Experimental Medicine*.

[11] Geusau A., Abraham K., Geissler K., Sator M. O., Stingl G., Tschachler E. (2001). Severe 2,3,7,8-tetrachlorodibenzo-p-dioxin (TCDD) intoxication: clinical and laboratory effects. *Environmental Health Perspectives*.

[12] Geusau A., Tschachler E., Meixner M., Päpke O., Stingl G., McLachlan M. (2001). Cutaneous elimination of 2,3,7,8-tetrachlorodibenzo-p-dioxin. *British Journal of Dermatology*.

[13] Całkosinski I., Rosińczuk-Tonderys J., Bronowicka-Szydełko A. (2013). Effect of tocopherol on biochemical blood parameters in pleuritis-induced rats treated with 2,3,7,8-tetrachlorodibenzo-p-dioxin. *Toxicology and Industrial Health*.

[14] Całkosiński I., Rosinczuk-Tonderys J., Dobrzynski M., Palka L., Bazan J. (2013). Occurrence of disseminated intravascular coagulation in 2,3,7,8-tetrachlorodibenzo-p-dioxin (TCDD)-induced pneumonia in the rat. *Advances in Experimental Medicine and Biology*.

[15] Li W., Matsumura F. (2008). Significance of the nongenomic, inflammatory pathway in mediating the toxic action of TCDD to induce rapid and long-term cellular responses in 3T3-L1 Adipocytes. *Biochemistry*.

[16] Wu D., Li W., Lok P., Matsumura F., Vogel C. F. A. (2011). AhR deficiency impairs expression of LPS-induced inflammatory genes in mice. *Biochemical and Biophysical Research Communications*.

[17] MacDonald C. J., Cheng R. Y. S., Roberts D. D., Wink D. A., Yeh G. C. (2009). Modulation of carcinogen metabolism by nitric oxide-aspirin 2 is associated with suppression of DNA damage and DNA adduct formation. *The Journal of Biological Chemistry*.

[18] MacDonald C. J., Ciolino H. P., Yeh G. C. (2004). The drug salicylamide is an antagonist of the aryl hydrocarbon receptor that inhibits signal transduction induced by 2,3,7,8-tetrachlorodibenzo-*p*-dioxin. *Cancer Research*.

[19] Ahmad N. S., Khalid B. A. K., Luke D. A., Nirwana S. I. (2005). Tocotrienol offers better protection than tocopherol from free radical-induced damage of rat bone. *Clinical and Experimental Pharmacology and Physiology*.

[20] Singh S. U. N., Casper R. F., Fritz P. C. (2000). Inhibition of dioxin effects on bone formation in vitro by a newly described aryl hydrocarbon receptor antagonist, resveratrol. *Journal of Endocrinology*.

[21] Całkosiński I. (2008). *The influence of tocopherol on diagnostic indexes of inflammatory reaction in rats undergoing dioxin exposition [Habilitation thesis]*.

[22] Li X., Johnson D. C., Rozman K. K. (1995). Effects of 2,3,7,8-tetrachlorodibenzo-p-dioxin (TCDD) on estrous cyclicity and ovulation in female Sprague-Dawley rats. *Toxicology Letters*.

[23] Li Y., Wang T., Wang P. (2011). Reduction of atmospheric polychlorinated dibenzo-*p*-dioxins and dibenzofurans (PCDD/Fs) during the 2008 Beijing olympic games. *Environmental Science and Technology*.

[24] Basselin M., Ramadan E., Chen M., Rapoport S. I. (2011). Anti-inflammatory effects of chronic aspirin on brain arachidonic acid metabolites. *Neurochemical Research*.

[25] Beal M. F. (2003). Mitochondria, oxidative damage, and inflammation in Parkinson's disease. *Annals of the New York Academy of Sciences*.

[26] Delwing D., Tagliari B., Chiarani F., Wannmacher C. M. D., Wajner M., de Souza Wyse A. T. (2006). *α*-Tocopherol and ascorbic acid administration prevents the impairment of brain energy metabolism of hyperargininemic rats. *Cellular and Molecular Neurobiology*.

[27] Fatokun A. A., Stone T. W., Smith R. A. (2007). Cell death in rat cerebellar granule neurons induced by hydrogen peroxide in vitro: mechanisms and protection by adenosine receptor ligands. *Brain Research*.

[28] Maharaj H., Maharaj D. S., Daya S. (2006). Acetylsalicylic acid and acetaminophen protect against oxidative neurotoxicity. *Metabolic Brain Disease*.

[29] Mandal P. K. (2005). Dioxin: a review of its environmental effects and its aryl hydrocarbon receptor biology. *Journal of Comparative Physiology B*.

[30] Takada Y., Bhardwaj A., Potdar P., Aggarwal B. B. (2004). Nonsteroidal anti-inflammatory agents differ in their ability to suppress NF-*κ*B activation, inhibition of expression of cyclooxygenase-2 and cyclin D1, and abrogation of tumor cell proliferation. *Oncogene*.

[31] Całkosiński I., Stańda M., Borodulin-Nadzieja L. (2005). The influence of 2,3,7,8-tetrachlorodibenzo-p-dioxin on changes of parenchymal organs structure and estradiol and cholesterol concentration in female rats. *Advances in Clinical and Experimental Medicine*.

[32] Całkosiński I., Rosińczuk-Tonderys J., Bazan J. (2013). The Influence of 2,3,7,8-tetrachlorodibenzo-p-dioxin (TCDD) on hematological parameters during experimentally induced pleuritis in rats. *Inflammation*.

[33] Paxinos G. T., Wotson C. (1986). *The Rat Brain in Stereotaxic Coordinates*.

[34] Całkosiński I., Borodulin-Nadzieja L., Wasilewska U. (2004). Effect of dioxins on reproduction in rats *in vivo*. *Advances in Clinical and Experimental Medicine*.

[35] Całkosiński I., Wasilewska U., Borodulin-Nadzieja L. (2004). Influence of 2,3,7,8-tetrachlorodibenzo-p-dioxin (TCDD) on the functioning and structure of ovaries and testicles in the offspring of rats. *Veterinary Medicine*.

[36] Genter M. B., Clay C. D., Dalton T. P., Dong H., Nebert D. W., Shertzer H. G. (2006). Comparison of mouse hepatic mitochondrial versus microsomal cytochromes P450 following TCDD treatment. *Biochemical and Biophysical Research Communications*.

[37] Jin M. H., Hong C. H., Lee H. Y., Kang H. J., Han S. W. (2008). Enhanced TGF-*β*1 is involved in 2,3,7,8-tetrachlorodibenzo-p-dioxin (TCDD) induced oxidative stress in C57BL/6 mouse testis. *Toxicology Letters*.

[38] Jin M. H., Hong C. H., Lee H. Y., Kang H. J., Han S. W. (2010). Toxic effects of lactational exposure to 2,3,7,8-tetrachlorodibenzo-p- dioxin (TCDD) on development of male reproductive system: involvement of antioxidants, oxidants, and p53 protein. *Environmental Toxicology*.

[39] Kakeyama M., Sone H., Tohyama C. (2008). Perinatal exposure of female rats to 2,3,7,8-tetrachlorodibenzo-p-dioxin induces central precocious puberty in the offspring. *Journal of Endocrinology*.

[40] Kloser E., Böhmdorfer S., Brecker L. (2011). Synthesis of 5-(Fluorophenyl)tocopherols as novel dioxin receptor antagonists. *European Journal of Organic Chemistry*.

[41] Oehme M., Biseth A., Schlabach M., Wiig O. (1995). Concentrations of polychlorinated dibenzo-p-dioxins, dibenzofurans and non-ortho substituted biphenyls in polar bear milk from Svalbard (Norway). *Environmental Pollution*.

[42] Oehme M., Schlabach M., Hummert K., Luckas B., Nordøy E. S. (1995). Determination of levels of polychlorinated dibenzo-p-dioxins, dibenzofurans, biphenyls and pesticides in harp seals from the Greenland Sea. *Science of the Total Environment*.

[43] Rosińczuk-Tonderys J. (2012). *Effect of dioxin on the structure of the central nervous system after tocopherol and acetylsalicylic acid administration in an experimental model [Habilitation thesis]*.

[44] Aksenova M. V., Aksenov M. Y., Mactutus C. F., Booze R. M. (2005). Cell culture models of oxidative stress and injury in the central nervous system. *Current Neurovascular Research*.

[45] Bharath S., Hsu M., Kaur D., Rajagopalan S., Andersen J. K. (2002). Glutathione, iron and Parkinson's disease. *Biochemical Pharmacology*.

[46] Halliwell B. (2006). Oxidative stress and neurodegeneration: where are we now?. *Journal of Neurochemistry*.

[47] Całkosiński I., Cegielski M., Dziegel P., Skalik R., Majda J. (2004). Apoptotic changes in the myocardium in the course of experimentally-induced pleurisy. *Folia Morphologica*.

[48] Gong N., Zhang M., Zhang X.-B., Chen L., Sun G.-C., Xu T.-L. (2008). The aspirin metabolite salicylate enhances neuronal excitation in rat hippocampal CA1 area through reducing GABAergic inhibition. *Neuropharmacology*.

[49] Hassoun E. A., Vodhanel J., Holden B., Abushaban A. (2006). The effects of ellagic acid and vitamin E succinate on antioxidant enzymes activities and glutathione levels in different brain regions of rats after subchronic exposure to TCDD. *Journal of Toxicology and Environmental Health Part A*.

[50] Lauritzen B., Lykkesfeldt J., Skaanild M. T., Angen Ø., Nielsen J. P., Friis C. (2003). Putative biomarkers for evaluating antibiotic treatment: an experimental model of porcine *Actinobacillus pleuropneumoniae* infection. *Research in Veterinary Science*.

[51] Kopf P. G., Scott J. A., Agbor L. N. (2010). Cytochrome P4501A1 is required for vascular dysfunction and hypertension induced by 2,3,7,8-tetrachlorodibenzo-p-dioxin. *Toxicological Sciences*.

[52] Lehtinen M. K., Bonni A. (2006). Modeling oxidative stress in the central nervous system. *Current Molecular Medicine*.

[53] Wang S.-J. (2006). Facilitatory effect of aspirin on glutamate release from rat hippocampal nerve terminals: Involvement of protein kinase C pathway. *Neurochemistry International*.

